# Predictive Value of the Tuberculin Skin Test among Newly Arriving Immigrants

**DOI:** 10.1371/journal.pone.0060130

**Published:** 2013-03-27

**Authors:** Christiaan Mulder, Brigit Mulleners, Martien W. Borgdorff, Frank van Leth

**Affiliations:** 1 KNCV Tuberculosis Foundation, The Hague, The Netherlands; 2 Center for Infection and Immunity Amsterdam (CINIMA), Academic Medical Center, University of Amsterdam, Amsterdam, The Netherlands; 3 Department of Infectious Diseases, Public Health Service, Amsterdam, The Netherlands; 4 Department of Clinical Epidemiology, Biostatistics and Bioinformatics, Academic Medical Center, University of Amsterdam, Amsterdam, The Netherlands; 5 Department of Global Health, Academic Medical Center, University of Amsterdam, Amsterdam Institute for Global Health and Development, Amsterdam, The Netherlands; McGill University, Canada

## Abstract

**Rationale:**

Screening and treating newly arriving immigrants for latent tuberculosis infection (LTBI) in low-incidence countries could be promising to reduce the tuberculosis incidence among this population. The effectiveness of screening with the tuberculin skin test (TST) is unknown.

**Objectives:**

To estimate the risk of progression to tuberculosis within two years after entry, stratified by TST result at entry.

**Methods:**

In a case-base design, we determined the prevalence of TST positives (10 mm and 15 mm) among a representative cohort of immunocompetent immigrants (n = 643) aged ≥18 years who arrived between April 2009 and March 2011 in the Netherlands (base cohort). Immigrants who progressed to tuberculosis within two years after arrival in 2005, 2006 or 2007 were extracted from the Netherlands Tuberculosis Register (case source cohort). The prevalence of TST positives from the base cohort was projected on the case source cohort to estimate the risk of progression to active tuberculosis by using Bayesian analyses to adjust for the sensitivity of the TST and Poisson regression analyses to take into account the random error of the number of extracted cases.

**Results:**

The prevalence of TST positives was 42% and 23% for a cut-off value of 10 mm and 15 mm, respectively. The overall risk of progression to tuberculosis if TST positive was 238 per 100,000 population (95% CI 151–343) and 295 per 100,000 population (95% CI 161–473) for a cut-off value of ≥10 mm and ≥15 mm, respectively. The corresponding risk for TST negatives was 19 (95% CI 0–59) and 58 (95% CI 25–103).

**Conclusion:**

The TST has the discriminatory ability to differentiate between individuals at low and high risk of disease.

## Introduction

In countries with a low-incidence of tuberculosis (TB), TB is primarily prevalent among first generation immigrants. In the Netherlands, 73% of the new patients with TB in 2010 were first generation immigrants, corresponding to an incidence of 45.6 per 100,000 persons, whereas this incidence was 1.6/100,000 for the native population [1]. Immigrants from high incidence countries have a high risk of developing TB in the first five years after immigration [2]. From the Netherlands Tuberculosis Register (NTR) it appeared that in 2010, 12% of the first generation immigrants with TB were diagnosed at entry, while another 25% were diagnosed with TB within 2.5 years after entry [1]. Furthermore, the majority of immigrants with TB had a unique strain of *Mycobacterium* TB (MTB), and the ones who belonged to a cluster (patients with identical DNA-fingerprint isolates) were often not epidemiologically linked [1]. These data suggest that immigrants acquire TB infection in their country of origin and that incident TB is a consequence of reactivation of latent TB infection (LTBI).

All immigrants aged >12 years from high–incidence countries intending to stay more than three months in the Netherlands, currently only undergo a mandatory screening for TB by chest x-ray (CXR) [3]. Immigrants are not screened for LTBI because the specificity of the tuberculin skin test (TST) is considered limited, due to cross-reactivity with bacille Calmette-Guérin (BCG) vaccination and environmental mycobacteria [4].

Interferon gamma release assays (IGRAs) were developed to improve the diagnosis of LTBI. These are based on identifying cellular production of interferon gamma (IFN-γ) in response to MTB specific antigens and do therefore not react on BCG and most environmental mycobactaria. One of those IGRAs is the QuantiFERON®-TB Gold In-Tube (QFT-GIT). In a recent study we have shown that immigrants with a positive QFT-GIT at entry had a considerable higher risk of progression to active TB within two years compared to immigrants with a negative QFT-GIT at entry [5]. This implies that targeted testing for LTBI, at least with the QFT-GIT, and subsequently offering preventive therapy, might contribute to decrease the incidence of TB.

The effectiveness of using the TST in screening immigrants for LTBI is still unknown since it has never been used in the Dutch setting. Several studies found that, among immigrant contacts of TB patients, the TST and QFT-GIT were both indicative in predicting the risk of developing TB [6], [7]. Kik et al. found a similar positive predictive value (PPV) for QFT-GIT and TST, irrespective whether a cut-off value for the TST of 10 mm or 15 mm was used. Also the negative predictive values of both tests were comparable [6]. The TST therefore might also have potential in screening immigrants for LTBI at entry, especially since the TST is relatively cheap, easy to administer, and extensively used in routine practice for many years. Whether the TST would be useful depends on its discriminatory ability in comparison with the QFT-GIT. The objective of this study was to predict the risk of progression to TB within two years after entry given the TST result at entry.

## Materials and Methods

### Ethics Statement

Ethical approval was obtained from the Netherlands Central Committee on Research Involving Subjects.

### Design

**Figure 1:**
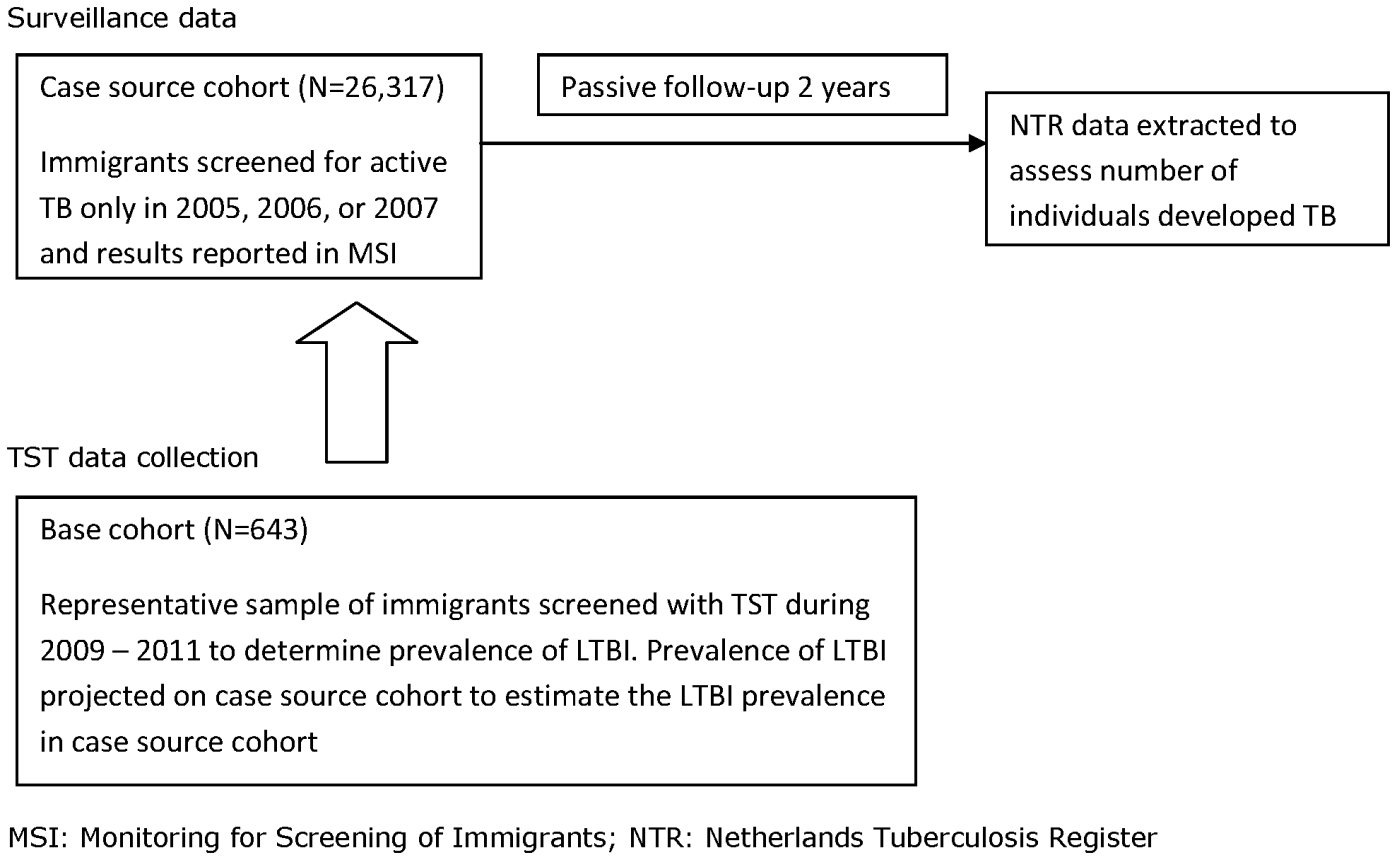
Schematic overview of study design.

This study is a post-hoc analysis of data derived from the study assessing the relationship between QFT-GIT result and risk of active TB in immigrants within two years of time of entry screening [5]. A case-base design was used, also labelled as ‘case-cohort’ design [8]. A representative group of controls is selected from which the cases originated, regardless of future disease status. A case-base design was used, because in a prospective design we would have needed a very large sample to obtain accurate risk estimates given the relatively low risk of progression to active TB [9]. The prevalence of TST positives (≥10 mm and ≥15 mm) was assessed in a representative sample of newly arriving immigrants at seven Public Health Services (PHSs) between April 2009 and March 2011, denoted as ‘base cohort’ (Figure 1). We projected this prevalence on a cohort of immigrants who were screened for active TB at arrival in 2005, 2006, or 2007 at the same seven PHS and were registered in the Monitoring for Screening of Immigrants (MSI), denoted as ‘case source cohort’. From this cohort the immigrants who developed TB within two years after arrival were extracted, denoted as ‘cases’, by matching the MSI with the NTR by sex, country of birth and date of birth. The TST status of the case source cohort was therefore “assessed indirectly” by assuming a similar distribution of TST test results as directly assessed in the base cohort. As a matter of fact, since the immigrants from the case source cohort were not tested for LTBI at entry, none of them were offered a course of preventive therapy.

All immigrants aged ≥18 years who visited the PHS for their entry screening, reported to be immunocompetent and provided written informed consent, were eligible for enrolment in the base cohort. We excluded immigrants who were diagnosed with active TB within six months after entry, because in the NTR these are reported as detected at entry screening and not during follow-up. Regarding the sample size, in our previous study 1570 immigrants consented. Out of these, 643 received a TST. The sample of 643 gave us enough power to find a prevalence of TST positives of 30% with a precision of 7%, and to do stratified analyses (sex, age, incidence in country of origin). The TST was performed according to the Mantoux method with 0.1 ml purified protein derivative RT23 (Statens Serum Institute, Copenhagen, Denmark). Experienced medical staff read the induration in millimetres after 48–120 hours (69% of the TST-results was read within 72 hours, 30% was read hereafter; the prevalence positive TST-results was similar for both reading times).

### Statistical Analyses

To calculate the risk of progression to active TB per 100,000 population we used a Bayesian approach with non-informative priors. The numerator (number of cases identified from the case source cohort) was modelled using the Poisson distribution to allow for uncertainties in the number of identified patients. The denominator (number of immigrants with positive/negative TST) took into account differences in sensitivity of the TST reported in the published data [6], [10], [11], [12], [13], [14]. The Bayesian model provided a posterior distribution for the risk of progression to active TB from which 20,000 random samples were drawn to arrive at a point estimate and 95% credibility interval (CI). We estimated the risk stratified by sex, age and incidence in country of origin (see Text S1 for more details).

The risk estimates of progression to active TB enabled us to calculate the numbers needed to treat (NNT), and the numbers needed to screen (NNS) to prevent one TB patient within two years, for TST ≥10 mm and TST ≥15 mm. We assumed a 60% efficacy rate of the preventive treatment [15].

Eligible patients could have 1 of 3 reasons for not being included in the present study. First, they could not have consented to participate in the original study; second, they did participate but did not want to have a TST administered, or third, they had a TST administered but there was no available result (test failure or no return for test reading). Given the post-hoc analysis, there is a need to carefully check for selection bias at each of these instances. We therefore compared the baseline characteristics of the base cohort with the case source cohort, the non-consenters and the participants without a TST result by Pearson χ^2^. Baseline characteristics were sex, age (18–24 years, 25–34 years and ≥35 years), region of origin (Europe & the Americas, North Africa & Middle East, Asia, Sub-Saharan Africa, Unknown), incidence of TB in country of origin (<100 (low), 100–199 (intermediate) and ≥200 (high) per 100,000 population), BCG vaccination (yes if BCG-scar was present and/or participant indicated being vaccinated), previous diagnosis of TB (yes/no), smoking (yes if ever smoked/no) and time in the Netherlands before screening (yes if ≤3 months in the Netherlands before screening/no). Associations were considered statistically significant when p-values were ≤0.05. Statistical analyses were conducted using SPSS 17.0 (Chicago, IL, USA) and WinBUGS 1.4.3 (Imperial College and MRC, UK).

## Results

**Figure 2:**
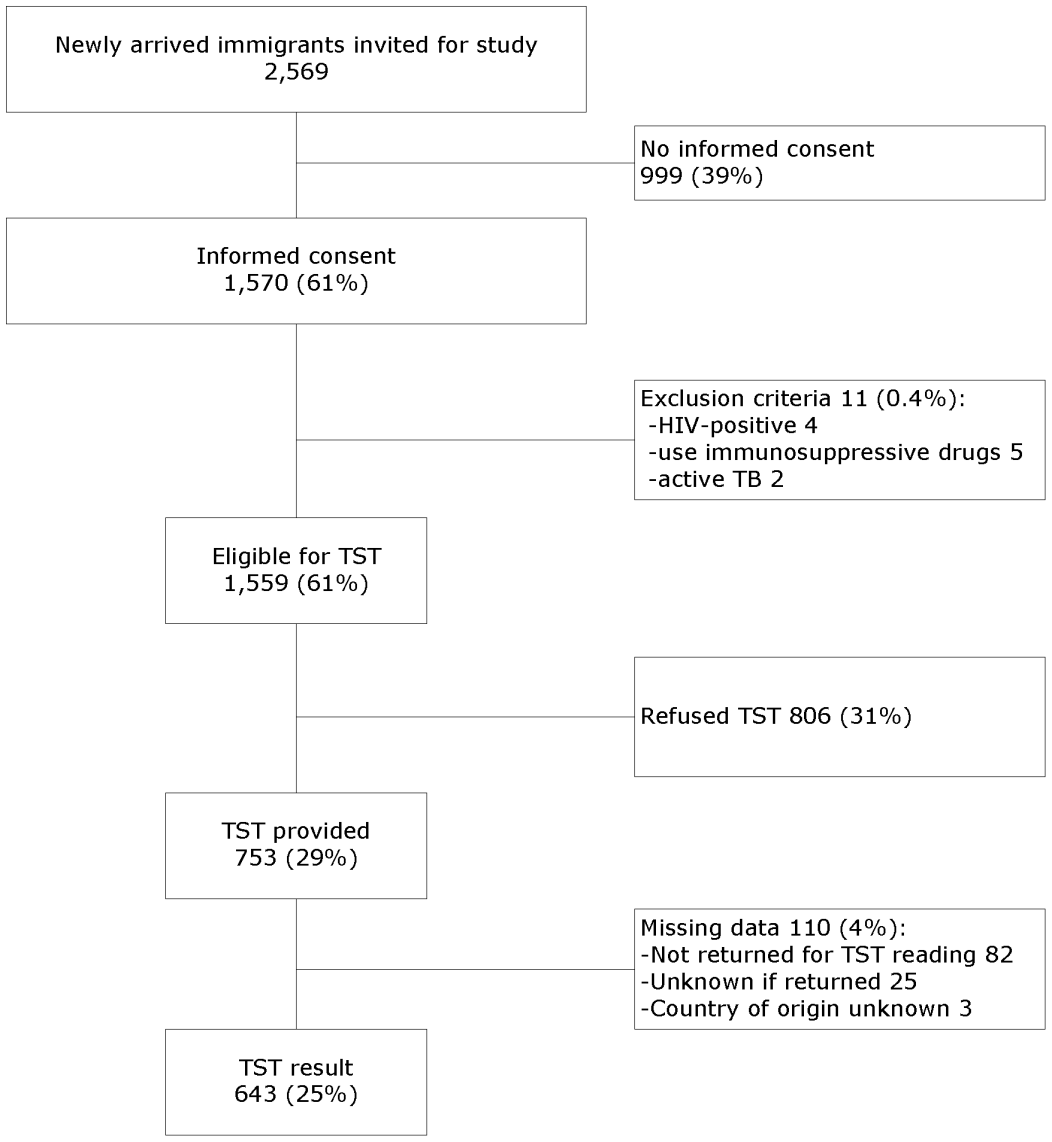
Flow diagram of study participants.

During the study period, 2,569 immigrants were approached for study enrolment at the seven PHSs. Out of 1,570 consenters, 1,559 were eligible for TST testing since 11 were excluded because they were HIV-positive (n = 4), used immunosuppressives (n = 5), or were diagnosed with active TB within 6 months (n = 2) (Figure 2). In 753 (48.3%) immigrants, out of those eligible, a TST was administered because others refused the TST due to a lack of time for the second visit. Out of these, 82 did not return for the reading of the TST, and in 25 the TST result was unknown because it was not registered. Three participants with no data on country of origin were excluded from all analyses, resulting in 643 (41%) immigrants with a TST result. The consenters were significantly more often from high-incidence countries than non-consenters (20% versus 14%, data not shown). Compared to the case source cohort, there were significantly more females (57% versus 52%), more immigrants from Asia (45% versus 41%), and more immigrants from high-incidence countries (23% versus 19%) in the base cohort (Table 1). The immigrants in the base cohort were significantly more often BCG-vaccinated (85% versus 76%) and from high incidence countries (23% versus 18%) than the immigrants without a TST result. Although the base cohort and the case source cohort differed in demographics a bit, we consider the base cohort representative for the case source cohort.

**Table 1:** Baseline characteristics for base cohort, case source cohort and participants without TST result.

	Base cohort†	Case source cohort*	Base cohort compared with case source cohort‡	No TST result	Base cohort compared with no TST result‡
	N (%)	N (%)	*P*-value	N (%)	*P*-value
Total	643 (100%)	26,317 (100%)	–	913 (100%)	–
Sex					
Female	370 (57%)	13,766 (52%)	0.020	488 (54%)	0.110
Male	273 (43%)	12,504 (48%)		425 (47%)	
Unknown	–	47 (0.2%)		–	
Age					
18 to 24 years	180 (28%)	7,877 (30%)	0.173	284 (31%)	0.072
25 to 34 years	306 (48%)	12,797 (49%)		449 (49%)	
≥35 years	157 (24%)	5,643 (21%)		180 (20%)	
Region of birth					
Europe & the Americas	156 (24%)	7,647 (29%)	0.014	245 (27%)	0.048
North Africa & Middle East	93 (15%)	3,680 (14%)		133 (15%)	
Asia	291 (45%)	10,849 (41%)		428 (47%)	
Sub-Saharan Africa	103 (16%)	3,894 (15%)		102 (11%)	
Unknown	–	247 (1%)		5 (0.5%)	
Incidence in country of origin					
0–99	302 (47%)	13,799 (52%)	0.001	470 (52%)	0.040
100–199	195 (30%)	7,231 (28%)		277 (30%)	
≥200	146 (23%)	5,040 (19%)		161 (18%)	
Unknown	–	247 (1%)		5 (0.5%)	
BCG vaccination					
Yes	549 (85%)	–	–	695 (76%)	<0.001
No	94 (15%)	–		59 (7%)	
Unknown	–			159 (18%)	
Previous diagnose of TB					
Yes	7 (1%)	–	–	12 (1%)	0.881
No	622 (97%)	–		879 (96%)	
Unknown	14 (2%)	–		22 (3%)	
Ever smoked					
Yes	190 (30%)	–	–	262 (29%)	0.390
No	450 (70%)	–		641 (70%)	
Unknown	3 (0.5%)	–		10 (1%)	
Time in NL before screening ≤3 months					
Yes	591 (92%)	–	–	840 (92%)	0.502
No	50 (9%)	–		65 (7%)	
Unknown	2 (0.3%)	–		8 (1%)	

BCG: bacille Calmette-Guérin; NL: the Netherlands, TST: tuberculin skin test.

† Sample of newly arriving immigrants at seven Public Health Services (PHSs) between April 2009 and March 2011.

* Three cohorts of immigrants who were screened at arrival in 2005, 2006 or 2007 and were registered in the Monitoring for Screening of Immigrants (MSI).

‡ Chi^2^ test.

Overall, 273 out of 643 (42%) were TST positive when the cut-off value was set at 10 mm and 145 (23%) when the cut-off value was set at 15 mm (Table 2).

**Table 2:** Prevalence of TST positives for cut-off value ≥10 mm and ≥15 mm.

	Total	Number of TST positives if ≥10 mm(% of total)	Number of TST positives if ≥15 mm(% of total)
Total	643	273 (42)	145 (23)
Sex			
Female	370	153 (41)	83 (22)
Male	273	120 (44)	62 (23)
Age (yr)			
18–24	180	50 (28)	20 (11)
25–34	306	148 (48)	86 (28)
≥35	157	75 (48)	39 (25)
Region of origin			
Europe & Americas	156	64 (41)	30 (19)
North Africa & Middle East	93	40 (43)	25 (27)
Other Asia	291	114 (39)	55 (19)
Sub-Saharan Africa	103	55 (53)	35 (34)
Incidence in country of origin			
<100	302	120 (40)	58 (19)
100–199	195	82 (42)	44 (23)
≥200	146	71 (49)	43 (29)
BCG-vaccinated			
Yes	549	249 (45)	128 (23)
No/unknown	94	24 (26)	17 (18)
Ever treated for TB			
Yes	7	5 (71)	3 (43)
No/unknown	636	268 (42)	142 (22)
Ever smoked			
Yes	190	95 (50)	45 (24)
No/unknown	453	178 (39)	100 (22)
≤3 months in NL before screening			
Yes	591	255 (43)	138 (23)
No/unknown	52	18 (35)	7 (14)

BCG: bacille Calmette-Guérin; NL: the Netherlands; TST: tuberculin skin test.

In the case source cohort we identified 30 immigrants who developed active TB within two years. Ten cases were clustered, but none were epidemiologically linked to a TB patient in the Netherlands according to the registries. When the TST cut-off value was set at 10 mm, the expected number of TST positives in the case source cohort based on the prevalence found among the base cohort, was 11,173 (42%) (Table 3). The median sensitivity of the TST was estimated at 90% (95% CI 72%–100%). The overall risk of progression to TB for TST positives was 238 per 100,000 population (95% CI 151–343) (Table 3). For TST negatives, this risk was 19 per 100,000 population (95% CI 0–59).

**Table 3:** Risk of progression to TB within two years after entry screening for TST ≥10 mm.

	Case source cohort	TB within two years*	Incidence of TBwithin two years per100.000 population	Expected TST positive at entry†	Estimated risk of progression toTB per 100.000 population‡	
	N	N		N (%)	TST-positive	TST-negative
Overall	26,317	30	114	11,173 (42)	238 (151–343)	19 (0–59)
Sex						
Female	13,766	15	109	5,692 (41)	233 (120–373)	17 (0–57)
Male	12,504	15	120	5,621 (45)	241 (122–388)	20 (0–66)
Age (yr)						
18–24	7,877	6	76	2,188 (28)	237 (75–472)	9 (0–36)
25–34	12,797	16	125	6,189 (48)	228 (122–364)	22 (0–74)
≥35	5,643	8	142	2,696 (48)	258 (99–476)	24 (0–89)
TB incidence in country of origin						
<100	13,799	12	87	5,483 (40)	193 (90–322)	13 (0–46)
100–199	7,231	12	166	3,041 (42)	346 (163–581)	26 (0–89)
≥200	5,040	6	119	2,451 (49)	212 (68–418)	20 (0–79)

TB: tuberculosis; TST: tuberculin skin test.

* Based on surveillance data from the MSI & NTR.

† Number estimated based on prevalence positive TST ≥10 mm in base cohort.

‡ Based on a median (95% CI) sensitivity for TST≥10 mm of 90% (72–100%).

For the cut-off value of 15 mm we estimated a sensitivity of 59% (95% CI 37%–82%). The overall risk of progression to TB for TST positives was 295 per 100.000 population (95% CI 161–473) (Table 4) and 58 per 100,000 population (95% CI 25–103) for TST negatives.

**Table 4:** Risk of progression to TB within two years after entry screening for TST ≥15 mm.

	Case source cohort	TB within two years*	Incidence of TBwithin two years per100.000 population	Expected TST positive at entry†	Estimated risk of progression toTB per 100.000 population‡	
	N	N		N (%)	TST-positive	TST-negative
Overall	26,317	30	114	5,934 (23)	295 (161–473)	58 (25–103)
Sex						
Female	13,766	15	109	3,088 (22)	281 (130–492)	55 (21–105)
Male	12,504	15	120	2,840 (23)	306 (139–538)	61 (23–116)
Age (yr)						
18–24	7,877	6	76	875 (11)	388 (108–830)	33 (7–75)
25–34	12,797	16	125	3,057 (24)	257 (122–447)	68 (26–130)
≥35	5,643	8	142	1,402 (25)	325 (114–643)	72 (21–157)
TB incidence in country of origin						
<100	13,799	12	87	2,650 (19)	261 (110–475)	42 (15–83)
100–199	7,231	12	166	1,632 (23)	423 (178–773)	83 (30–166)
≥200	5,040	6	119	1,483 (29)	228 (64–486)	64 (14–149)

TB: tuberculosis; TST: tuberculin skin test.

* Based on surveillance data from the MSI & NTR.

† Number estimated based on prevalence positive TST ≥15 mm in base cohort.

‡ Based on a median (95% CI) sensitivity for TST≥15 mm of 59% (37–82%).

For both cut-off values of the TST, no marked differences in risk of progression to TB were estimated between the males and females., while minor differences were seen with respect to strata of age (15 mm) or TB-incidence in country of origin (10 mm and 15 mm)s (Tables 3, 4).

The NNT and NNS to prevent one TB patient within two years after entry for a TST cut-off value of 10 mm were 700 and 1649, respectively. The corresponding NNT and NNS for a TST cut-off value of 15 mm were 565 and 2505, respectively.

## Discussion

This study shows that the TST had the discriminatory ability to identify high and low risk groups for progression to active TB among newly arriving immigrants. This ability was irrespective of the cut-off value of 10 mm or 15 mm, but did not apply for the highest age group (if cut-off value 15 mm) and highest incident category with respect to country of origin (for both cut-off values). The estimated risks for TST positives to progress to TB within two years were considerably higher than the incidence of 50/100,000 which is used in the Netherlands as a cut-off value to define a risk group. Appropriate preventive interventions to lower the incidence of TB among newly arriving immigrants should therefore be undertaken.

In a previous study [5] we found that the risk of progression to active TB within two years per 100,000 population was 467 (95% CI 314–603) among QFT-GIT positive individuals whereas the risk of progression to active TB among QFT-GIT negatives was 25 (95% CI 0–64). It appears therefore that the discriminatory ability of QFT-GIT is slightly better than that of the TST using either a 10 mm or 15 mm cut-off value. The discriminatory ability of TST was somewhat lower than of QFT-GIT, probably because of the, in general, lower specificity (cross reactions attributable to BCG and environmental mycobacteria) and, when using a 15 mm cut-off value, a lower sensitivity to identify LTBI. However, because most credibility intervals overlap these differences between the tests should be interpreted carefully.

Screening immigrants at entry will only be effective if positive test results are followed by adequate interventions like prescribing preventive treatment. The Dutch preventive treatment regimen is three months daily isoniazid plus rifampicin, or six months daily isoniazid. The duration and the risk of severe side effects, such as hepatotoxicity, influence adherence and could harm the efficacy of the treatment [16]. The NNT to prevent one TB patient within two years after entry we have presented when screening with the TST was considerably higher (for both cut-off values) than the NNT of 350 we have reported previously for the QFT-GIT [5]. The corresponding NNS when screening with TST was also higher (for cut-off value 15 mm) than the NNS of 1800 we have reported for the QFT-GIT. These findings suggest that screening newly arriving immigrants for LTBI by QFT-GIT would be more effective than screening with TST. It has been proposed that screening individuals may be most cost-effective if TST positives are subsequently tested with QFT-GIT [17], [18], [19], but more cost-effectiveness studies are needed in screening immigrants, especially by comparing strategies including screening with QFT-GIT, TST, or a combination of these diagnostics. Even if screening immigrants with TST is cost-effective, it will be logistically challenging to have all immigrants visit the PHS twice for placing and reading of the TST.

We did not observe marked differences in the risk of progression to disease for both TST-positives and TST-negatives between the strata of sex, age and incidence in country of origin. The overlapping credibility intervals are a result of the small number of immigrants who progressed to disease.

There were several limitations of this study. Firstly, more than half of the immigrants eligible for TST testing refused. However, there were no marked differences in baseline characteristics between the participants and the ones who refused. The main reason for refusing was a lack of time for a second visit and the fact that it was not mandatory. This highlights that the willingness of immigrants to become screened by TST is limited. There is some controversy whether physician’s and patient’s adherence to guidelines is determined by the diagnostics used. Grinsdale et al. showed that contacts of culture-confirmed TB patients were more likely to complete evaluation and to complete isoniazid preventive therapy when tested with IGRA compared to the TST [20], whereas Shah et al. observed no differences in treatment initiation or completion between the period pre- and post implementation of the IGRA [21]. Secondly, although we aimed to measure the prevalence of TST-positives in a representative cohort, we observed, be it relatively small, demographic differences between the base cohort and the case source cohort. A consequence of having a slight overrepresentation of immigrants originating from high incidence countries in the base cohort might be that we have overestimated the overall prevalence of TST positives, and thereby underestimated the overall risk of progression to disease. Thirdly, we had to assume that the immigrants who progressed to disease within two years were already infected at entry. The absence of clustering and the absence of confirmed contact among clustered cases are consistent with reactivation of LTBI. Fourthly, we could not adjust for potential bias as a result of differential drop-out of immigrants during the two year passive follow-up. In other words, from the surveillance data we could not determine whether diseased individuals were more prone to (re)migrate or stay in the host country. Finally, our findings cannot be translated to newly arriving immigrant children (<18 years).

In conclusion, in this study we found that newly arriving immigrants with a positive TST result at entry were at considerable risk of progression to active TB, whereas for TST negatives this risk was limited. Nevertheless, the discriminatory ability of the TST was somewhat lower than that of the QFT-GIT. This suggests that the QFT-GIT, either used alone or in combination with the TST, is the favorable diagnostic tool for screening newly arriving immigrants coming to low-incidence countries. Further study is needed with respect to the cost-effectiveness for the TST and the QFT-GIT in the immigrant screening program to gain further evidence for considering screening immigrants for LTBI.

## Supporting Information

Text S1 **Calculation of the risk of progression to tuberculosis.**
(DOC)
