# Supplementary material for: Predictive Value of the Tuberculin Skin Test among Newly Arriving Immigrants
Source: PLoS One. 2013 Mar 27;8(3):e60130. doi: 10.1371/journal.pone.0060130 (PMC3609741; doi:10.1371/journal.pone.0060130)
Supplement: Text S1 — Calculation of the risk of progression to tuberculosis. (DOC) [file pone.0060130.s001.doc]

Online Supporting Information

To calculate the risk of progression to TB we used Poisson regression analyses to take into account the random error of the number of extracted cases. We adjusted for the sensitivity of the TST (for both cut-off values) for detecting those individuals who progressed to TB based on previous published data [1,2,3,4,5,6]. From each of these studies we included the reported sensitivity and precision (the inverse of the squared standard error of the sensitivity estimate) for the prediction of TB in TST positive individuals in a Bayesian model to obtain a posterior distribution of the sensitivity for the TST [7]. This model contained a random error term to denote the different studies included. The prior distributions for both the mean and the precision of the sensitivity were non-informative. Twenty thousand random draws from the resulting posterior distribution were then projected on the total number of patients with disease progression within the case source cohort. This resulted in a median and 95% credibility interval (95% CI) for the risk of progression to TB for specified strata.

We used 2,000 iterations for the burn-in period of the model. The iterations for estimation were repeated in batches of 5,000 until the Monte-Carlo (MC) errors of all the estimates were below the conventional 1% of the standard error of the estimate. Convergence was assessed by exploring the trace plots and the density plots of the estimates, and by ‘mixing of the estimates’ of two different chains with markedly different initial values.

Convergence was achieved based on all three assessments. The median of the posterior distribution of the sensitivity was 90% (95% CI 72%-100%) when the TST cut-off value was 10 mm and 59% (95% CI 37%-82%) when the cut-off value was 15 mm.

**References**

1. Hill PC, Jackson-Sillah DJ, Fox A, Brookes RH, de Jong BC, et al. (2008) Incidence of tuberculosis and the predictive value of ELISPOT and Mantoux tests in Gambian case contacts. PLoS One 3: e1379.

2. Torres Costa J, Silva R, Sa R, Cardoso MJ, Nienhaus A (2011) Serial testing with the interferon-gamma release assay in Portuguese healthcare workers. Int Arch Occup Environ Health 84: 461-469.

3. Harstad I, Jacobsen GW, Heldal E, Winje BA, Vahedi S, et al. (2010) The role of entry screening in case finding of tuberculosis among asylum seekers in Norway. BMC Public Health 10: 670.

4. Diel R, Loddenkemper R, Meywald-Walter K, Niemann S, Nienhaus A (2008) Predictive value of a whole blood IFN-gamma assay for the development of active tuberculosis disease after recent infection with Mycobacterium tuberculosis. Am J Respir Crit Care Med 177: 1164-1170.

5. Kik SV, Franken WP, Mensen M, Cobelens FG, Kamphorst M, et al. (2010) Predictive value for progression to tuberculosis by IGRA and TST in immigrant contacts. Eur Respir J 35: 1346-1353.

6. Winje BA, Oftung F, Korsvold GE, Mannsaker T, Jeppesen AS, et al. (2008) Screening for tuberculosis infection among newly arrived asylum seekers: comparison of QuantiFERONTB Gold with tuberculin skin test. BMC Infect Dis 8: 65.

7. Ntzoufras I (2009) Basyesian Modeling Using WinBUGS New York: Wiley.
